# Prevalence Rate and Risk Factors of Depression in Outpatients with Premature Ejaculation

**DOI:** 10.1155/2013/317468

**Published:** 2013-06-15

**Authors:** Xiansheng Zhang, Jingjing Gao, Jishuang Liu, Lei Xia, Jiajia Yang, Zongyao Hao, Jun Zhou, Chaozhao Liang

**Affiliations:** Department of Urology, The First Affiliated Hospital of Anhui Medical University, Hefei, Anhui 230032, China

## Abstract

The purpose of this study is to investigate the prevalence rate and risk factors of depression in outpatients who were diagnosed with PE. Therefore, between September 2009 and September 2011, 1801 outpatients at andrology clinics were enrolled and consented to participate in our survey by completed a verbal questionnaire. It included the following: (1) demographic data (e.g., age, body mass index), (2) PE duration, medical history, and sexual history, (3) self-estimated intravaginal ejaculatory latency times, (4) the Zung Self-rating Depression Scale (SDS), and (5) the National Institute of Health Chronic Prostatitis Symptom Index (NIH-CPSI) and (6) the International Index of Erectile Function (IIEF-5). The results showed that a total of 1,206 patients were diagnosed with PE. The prevalence rate of depression in these PE patients was 26.78%. Depression was associated with PE duration, NIH-CPSI score, and IIEF-5 score. Risk factors for depression specifically included PE durations for 13–24, 25–60, or ≥61 months, CPSI scores of 15–30 or ≥31, and IIEF-5 scores <22. These findings suggested that several associated factors (PE duration, CPSI scores, and IIEF-5 scores) were the risk factors of depression in men with PE.

## 1. Introduction 

Premature ejaculation (PE) is a common male sexual dysfunction, which affects 20~30% of the male population [1–3]. Previous studies have shown that PE was associated with a range of negative psychological effects, including anxiety, depression, and distress in men and their female partners. Negative psychological effects might play an important role in precipitating or maintaining PE and seriously impair male health and couples' sexual relationships [4–6].

In a large observational study conducted by Patrick et al., men with PE reported a greater level of distress than men without PE (2.69 versus 0.69; 5-point scale ranging from 0 = not all distressed to 4 = extremely distressed) [7]. Further analysis of the same survey showed that, relative to men without PE, men with PE had lower levels of sexual functioning and satisfaction as well as higher levels of personal distress and interpersonal difficulty [8]. Another survey of 334 Korean men revealed a significant relationship between PE and depression. Compared with control (no PE) group, men with self-assessed PE suffered from various psychological problems, such as depression, low self-esteem, and sexual satisfaction [4].

Although putative negative psychological effects of PE have been discussed in recent years, correlations between PE and negative psychological effects have not been clarified, especially in China. In 2005, Lau et al. [9] investigated 298 young married Chinese men and confirmed that PE was significantly associated with mental health disturbances. However, that study did not include an in-depth examination of the factors relating the association. Therefore, in our survey, we determined the prevalence rate of and risk factors for depression in men who sought treatment for the complaint of ejaculating prematurely.

## 2. Subjects and Methods

### 2.1. Subjects

From September 2009 to September 2011, a noninterventional, observational, cross-sectional field survey was conducted. Patients who sought treatment for the complaint of ejaculating prematurely were enrolled from andrology clinics located at three cities (namely, Hefei city, Suzhou city, and Wangjiang city) in Anhui province, China. This study were evaluated and approved by the Anhui Medical University Research Subject Review Board.

Subjects had to meet the following criteria to be included in this study: (1) age ≥18 years; (2) heterosexual man in a stable, monogamous sexual relationship for at least 6 months; (3) able to speak Chinese. Each subject's medical history was carefully evaluated to rule out mental and/or other major medical diseases. Patients on medications that may affect ejaculatory function, such as antidepressants or phosphodiesterase type 5 inhibitors, were excluded.

### 2.2. Study Design and Procedure

Prior to study enrollment, all patients were informed about this survey. A careful medical and sexual history was also taken by an experienced clinician. Eligible patients were asked to provide written consent. Prior to survey administration, a presurvey was given to a small sample of subjects to modify the originally designed items to ensure that the questionnaire was comprehensive and easily understood.

This survey was conducted by face-to-face interviews. All subjects participated in the survey by completing a verbal questionnaire, which collected the following data: (1) age, body mass index (BMI), educational level, and occupation, (2) PE duration, medical and sexual history, (3) self-estimated intravaginal ejaculatory latency times (IELTs), (4) the Zung Self-rating Depression Scale (SDS), and (5) the National Institute of Health Chronic Prostatitis Symptom Index (NIH-CPSI) and International Index of Erectile Function (IIEF-5). The reliability of the instruments was assessed using Cronbach's alpha coefficient. The internal consistencies of the instruments (SDS, NIH-CPSI, and IIEF-5) were 0.80, 0.77, and 0.81, respectively.

Based on ISSM criteria [10], a patient was diagnosed with PE if they experienced vaginal penetration for less than one minute, a loss of control, and/or negative sexual consequences. Following the classification of diagnosis, PE patients were then divided into two subgroups: (1) lifelong PE group, who suffered from PE since the first sexual experience and (2) acquired PE group, who experienced normal vaginal ejaculation, although early ejaculation had occurred at some point in their life [11].  Table 1 provides the diagnostic criteria for lifelong and acquired PE. 

Depression was assessed by the SDS [12]. Both of these questionnaires consisted of 20 questions with a specific scoring system. The specific scoring system was applied in which each question had four possible answers, which were individually assigned 1, 2, 3, or 4 points (1 = rarely, 2 = occasionally, 3 = frequently, and 4 = always). After the questionnaire was completed, the total score for the SDS was combined, divided by 80, and then compared to a standard cutoff score for depression. A standard cut-off of 0.5 was employed such that a score <0.5 indicated no depression and a score ≥0.5 confirmed depression.

The NIH-CPSI is a reliable, convenient, self-administered index that is widely used across scientific research and clinical studies. The assessment measures chronic prostatitis (CP) symptoms and their impact on daily life [13]. According to NIH-CPSI scores, CP symptoms can be separated into mild (0–14), moderate (15–30), or severe (≥31) categories. 

The IIEF-5 questionnaire contained five questions that were answered according to symptoms (scale range 0–5). IIEF-5 scores ≥22 points were rated as normal erectile function, while scores <22 points were rated as erectile dysfunction (ED).

### 2.3. Statistical Analysis

Collected data were analyzed by SPSS (SPSS Inc., v. 13.0, Chicago, IL, USA). The relationship between depression and associated factors in PE men was examined by the chi-square test. Multiple logistic regression was used to evaluate the risk factors of depression. Odds ratios (ORs) and 95% confidence intervals (CIs) were calculated to examine association strength. Statistical significance was defined as *P* < 0.05.

## 3. Results

Of 2,513 outpatients who met the inclusion criteria, 1,801 completed the questionnaire (425 rejected participation; 287 provided incomplete questionnaires), yielding a response rate of 71.67%. Ultimately, 1206 (66.96%, 1206/1801) patients were diagnosed with PE based on ISSM criteria. Among those 1206 men, 483 (40.05%) patients had lifelong PE, and 723 (59.95%) had acquired PE. The mean patient age, BMI, and PE duration were 35.23 ± 11.45 years, 26.12 ± 3.18 kg/m^2^, and 32.15 ± 20.52 months, respectively. Detailed demographic information of enrolled men with PE is summarized in Table 2.

According to the SDS, 323/1206 (26.78%) patients experienced depression. Based on the diagnostic criteria of ED (IIEF-5 scores <22), 813/1206 (67.41%) patients in the PE group were diagnosed with ED. Chi-square test results showed that depression in PE patients was significantly associated with PE duration (*X* ^2^ = 17.425, *P* = 0.001), NIH-CPSI score (*X* ^2^ = 44.500, *P* < 0.001), and IIEF-5 score (*X* ^2^ = 8.697, *P* = 0.003). The following factors did not differ between depressed and not depressed men: age (*X* ^2^ = 0.496, *P* = 0.920), BMI (*X* ^2^ = 0.087, *P* = 0.768), educational status (*X* ^2^ = 0.749, *P* = 0.862), occupational status (*X* ^2^ = 2.022, *P* = 0.918), and PE subtype (*X* ^2^ = 0.506, *P* = 0.477) (Table 3). As reported in Table 4, multiple logistic regression showed that risk factors for depression among patients with PE included PE duration of 13–24 months (OR 1.42, CI 1.15–3.12), 25–60 months (OR 2.08, CI 1.33–4.97), or ≥61 months (OR 4.02, 95% CI 3.85–10.47), NIH-CPSI score of 15–30 (OR 2.01, 95% CI 1.23–4.15) or ≥31 (OR 4.98, 95% CI 3.87–11.21), and IIEF-5 score <22 (OR 4.11, 95% CI 3.43–9.15).

## 4. Discussion

Several studies have shown that men with PE may be influenced by negative psychological effects [8, 9, 14]. Negative psychological effects of PE can be displayed as personal distress, anxiety, and depression, which can impair physical and mental health of PE patients and their partners [15]. Indeed, a poor sexual relationship can produce feelings of frustration for both partners in a relationship [16, 17].

In the current study, we found that 26.78% patients with PE suffered from depression. In addition, 25.67% of lifelong PE patients and 27.52% of acquired PE patients were diagnosed with depression. However, there was no significant correlation between PE subtype and depression. Moreover, the results of this study also showed that PE was not significantly associated with common demogrphaic factors, including age, BMI, educational status, and occupational status, suggesting that these factors likely do not influence the occurrence of depression in PE patients. 

However, depression was significantly associated with PE duration. Specifically, multiple logistic regression indicated that PE durations extending beyond a year (13–24 months, 25–60 months, or ≥61 months) elevated risk of depression in PE patients. Considering the work of Graziottin and Althof [16], we thought that the relationship between PE duration and depression may best be explained as follows. Initially, the woman may keep silent to avoid hurting the man's feelings. Then, she and her partner may discuss reasons for losing sexual desire and/or failing to reach sexual climax. A continuous change in the woman's attitude across the duration of PE may increase the man's negative psychological effects, such as depression. Hence, in this way greater duration of PE may create or enhance emotional and physical dissatisfaction between partners.

It has been suggested that CP symptoms may be an important organic cause of PE [18–20]. Mehik et al. [21] showed that from 26.2% to 42.5% of men with CP (based on NIH-CPSI score) or chronic pelvic pain syndrome experienced PE. In our survey, we discovered that the prevalence rate of depression was significantly associated with NIH-CPSI scores such that the higher the CPSI score, the more likely that the patient experienced depression. Further analyses revealed that NIH-CPSI scores above 15 were a distinct risk factor of depression in PE patients. Based upon these results, we speculated that prostatitis symptoms negatively affected a patients' mood, which might then induce a psychological burden and aggravate emotional and physical pain in PE patients.

A similar relationship was also identified between depression and ED, as demonstrated by IIEF-5 scores. Compared to patients with PE who have normal erectile function, men suffering from both PE and ED may be more vulnerable to be affected by depression. Indeed, IIEF-5 scores less than 22 were the risk factors of depression in PE patients. Our findings support Buvat [22] who suggested that PE and ED may derive from a vicious cycle. As PE patients repeatedly attempt to reduce levels of excitation, in order to delay ejaculation, negative psychological effects may be induced. Consequently, the patient experiences loss of erectile function. Therefore, this vicious cycle will, in the long term, exacerbate negative psychological effects.

The current study has several limitations that require consideration. (1) Our survey sample included patients who presented complaints of PE and sought treatment. However, patients with PE complaints not referred to andrology clinics were not enrolled in our study. As a result, there may be some discrepancies between our findings and the true rate of depression among patients with PE. (2) We collected patient information by face-to-face interviews, which may have made some patients embarrassed due to the sensitive, personal nature of the research. While we believe that it is important to interact with patients, we certainly acknowledge that others methods (e.g., internet-based survey) can be used for data collection. (3) There is no control group of non-PE men in the study, making the comparisons with nonaffected populations difficult. Further research that includes comparative control groups should be conducted.

In conclusion, PE is a multifactored sexual dysfunction that is associated with negative psychological effects, which seriously impairs patient health and sexual relationships. In our survey, we found a substantial prevalence of depression among patients with PE (26.78%). The prevalence was further elevated among patients with long-term PE, high NIH-CPSI score, and low IIEF-5 score. These variables were all risk factors for depression.

## Figures and Tables

**Table 1:** Diagnostic criteria for lifelong and acquired PE.

Lifelong PE	Acquired PE
(1) Ejaculation occurs too early at nearly every intercourse (2) Occurs with (nearly) every woman (3) Occurs from about the first sexual encounter onwards (4) Occurs in the majority of cases within 30 s (70%), within 60 s (90%), or between 1 and 2 min (10%) (5) Remains rapid during life (70%) or becomes aggravated with aging (30%) (6) The ability to delay ejaculation (i.e., withhold ejaculation at the moment of imminent ejaculation) may be diminished or lacking	(1) Ejaculation occurs too early at some point in life (2) Normal ejaculation experiences prior to the start of problems (3) Occurs either suddenly or with a gradual onset (4) Occurrence may be associated with the following (i) Urological dysfunction (e.g., erectile dysfunction or prostatitis) (ii) Thyroid dysfunction (iii) Psychological or relationship problems (5) The ability to delay ejaculation (i.e., withhold ejaculation at the moment of imminent ejaculation) may be diminished or lacking

**Table 2:** Demographic characteristics of men with PE.

Characteristics	Subjects		Lifelong PE		Acquired PE	
	(*N* = 1206)		(*N* = 483)		(*N* = 723)	
	*N *	%	*N *	%	*N *	%
Age, years						
20–29	489	40.55	339	70.19	150	20.75
30–39	372	30.85	86	17.81	286	39.56
40–49	178	14.76	25	5.18	153	21.16
50–66	167	13.85	33	6.83	134	18.53
Mean ± SD	35.23 ± 11.45		28.86 ± 6.23		39.48 ± 12.15	
BMI, kg/m^2^						
≤24	475	39.39	203	42.03	272	37.62
>24	731	60.61	280	57.97	451	62.38
Mean ± SD	26.12 ± 3.18		24.17 ± 3.12		27.42 ± 4.14	
Duration, months						
0–12	195	16.17	121	25.05	74	10.24
13–24	367	30.43	241	49.90	126	17.43
25–60	412	34.16	100	20.70	312	43.15
≥61	232	19.24	21	4.35	211	29.18
Mean ± SD	32.15 ± 20.52		27.22 ± 13.47		37.45 ± 19.23	
Educational status						
Primary and illiterate	51	4.23	31	6.42	20	2.77
Secondary school	566	46.93	218	45.13	348	48.13
College	519	43.03	180	37.27	339	46.89
Postgraduate and above	70	5.80	54	11.18	16	2.21
Occupational status						
Workers	365	30.27	102	21.12	263	36.38
Students	115	9.54	92	19.05	23	3.18
Drivers	152	12.60	64	13.25	88	12.17
Farmers	71	5.89	41	8.49	30	4.15
Officials	132	10.95	83	17.18	49	6.78
Businessmen	144	11.94	58	12.01	86	11.89
Other occupations	227	18.82	43	8.90	184	25.45

Subjects: men with PE; SD: standard deviation.

**Table 3:** Factors associated with depression in men with PE.

Factors (df)^a^	With depression		Without depression		Total	[*X* ^2^], *P * value
	(*N* = 323)		(*N* = 883)		( *N* = 1206)
	*N *	%	*N *	%	*N *
Age, years (3)						
20–29	127	25.97	362	74.03	489	**[0.496],** **0.920**
30–39	100	26.88	272	73.12	372	
40–49	48	26.97	130	73.03	178	
50–66	48	28.74	119	71.26	167	
BMI, kg/m^2^ (1)						
≤24	125	26.32	350	73.68	475	**[0.087],** **0.768**
>24	198	27.09	533	72.91	731	
Educational status (3)						
Primary and/or illiterate	15	29.41	36	70.59	51	**[0.749],** **0.862**
Secondary school	147	25.97	419	74.03	566	
College	140	26.97	379	73.03	519	
Postgraduate and above	21	30.00	49	70.00	70	
Occupational status (6)						
Workers	103	28.22	262	71.78	365	**[2.022],** **0.918**
Students	28	24.35	87	75.65	115	
Drivers	39	25.66	113	74.34	152	
Farmers	16	22.54	55	77.46	71	
Officials	39	29.55	93	70.45	132	
Businessmen	38	26.39	106	73.61	144	
Other occupations	60	26.43	167	73.57	227	
Subtype (1)						
Lifelong PE	124	25.67	359	74.33	483	**[0.506],** **0.477**
Acquired PE	199	27.52	524	72.48	723	
Duration, months (3)						
0–12	39	20.00	156	80.00	195	**[17.425],** **0.001**
13–24	83	22.62	284	77.38	367	
25–60	119	28.88	293	71.12	412	
≥61	82	35.34	150	64.66	232	
NIH-CPSI, scores (2)						
0–14	145	20.80	552	79.20	697	**[44.500],** **<0.001**
15–30	147	32.38	307	67.62	454	
≥31	31	56.36	24	43.64	55	
IIEF-5, scores (1)						
≥22	84	21.37	309	78.63	393	**[8.697],** **0.003**
<22	239	29.40	574	70.60	813	

^a^Degrees of freedom (df) for *X* ^2^ test, *N* = 1206 in all cases.

**Table 4:** Risk factors revealed by multiple logistic regression for depression in men with PE.

Risk factors	Significance	Odds ratio	95% confidence interval	
			Lower	Upper
Duration, months				
0–12	1.00			
13–24	0.01	1.42	1.15	3.12
25–60	0.00	2.08	1.33	4.97
≥61	0.00	4.02	3.85	10.47
NIH-CPSI, scores				
0–14	1.00			
15–30	0.00	2.01	1.23	4.15
≥31	0.00	4.98	3.87	11.21
IIEF-5, scores				
≥22	1.00			
<22	0.00	4.11	3.43	9.15
